# Rare case of thrombolysis for ischemic stroke with a concurrent perforated duodenal ulcer

**DOI:** 10.1093/jscr/rjaf886

**Published:** 2025-11-11

**Authors:** Rositsa Gancheva Krasteva, Tsanko I Yotsov, Kristina K Stancheva

**Affiliations:** Department of Neurology, University Multiprofile Hospital for Active Treatment MEDIKA, Ruse 7000, Bulgaria; Department of Health Care, University of Ruse “Angel Kanchev”, Ruse 7000, Bulgaria; Department of Health Care, University of Ruse “Angel Kanchev”, Ruse 7000, Bulgaria; Department of Surgery, University Multiprofile Hospital for Active Treatment MEDIKA, Ruse, Bulgaria; Department of Neurology, University Multiprofile Hospital for Active Treatment MEDIKA, Ruse 7000, Bulgaria

**Keywords:** acute ischemic stroke, perforated peptic ulcer venous thrombolysis, tenecteplase

## Abstract

Acute ischemic stroke (AIS) is associated with an increased risk of mortality in surgical patients and its management is a clinical challenge. We present a case of a patient with AIS and concomitant perforated peptic ulcer, and venous thrombolysis was performed. Due to persistent abdominal pain, computed tomography was performed, showing free abdominal gas, and open surgery revealed duodenal perforation, which was corrected by excision, Mikulitz pyloroplasty, and Cellan-Jones patch. Several complications were observed in the postoperative period, which were treated conservatively, without lasting consequences. At follow-up, the patient regained almost full motor function and had no complaints regarding the repaired perforation. Venous thrombolysis is an effective treatment for AIS, but no cases have been described in surgical patients. This case represents an unconventional, simultaneous approach to the treatment of both initial conditions. Both treatment methods—thrombolysis and surgical procedure—can be successfully applied in selected cases.

## Introduction

Acute ischemic stroke (AIS) is associated with an increased risk of mortality among surgical patients and treatment of AIS in patients requiring surgery presents a clinical challenge due to the increased risk of postoperative bleeding. Venous thrombolysis has been shown to be an effective treatment for AIS, but its use in surgical patients remains controversial due to concerns about bleeding.

## Case report

A 69-year-old woman presented with abdominal pain of 2 weeks' duration. She was seen by a rural general practitioner (GP) and referred to hospital for further evaluation without evidence of stroke. The patient's medical history included well-controlled hypertension, a coiled cerebral aneurysm, and a smaller one, left for follow-up, spinal stabilization at L1 to L3 levels, and transanal resection of a high-grade dysplastic polyp.

**Figure 1:**
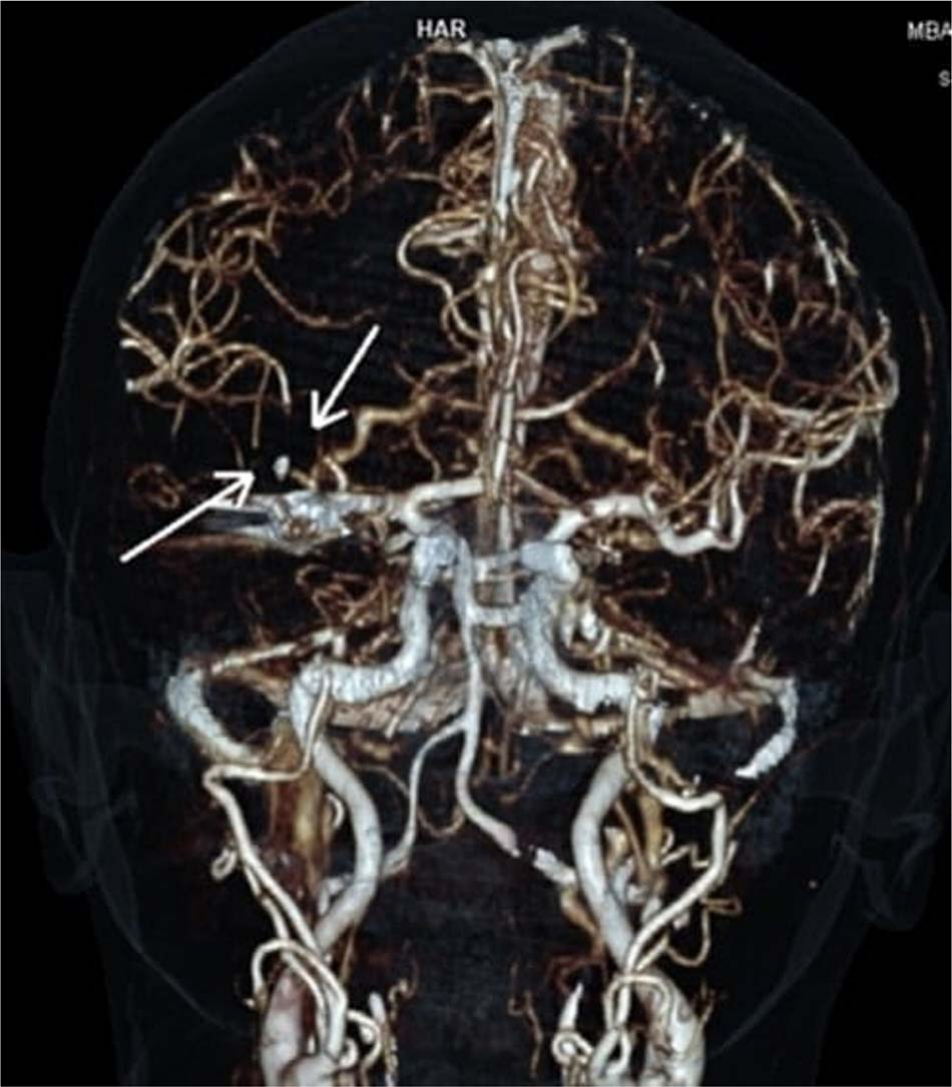
CTA reconstruction indicating proximal occlusion of M2 segmental branches of MCA (arrows).

On arrival at the hospital emergency department, ~2 h after the GP visit, moderate-to-severe left hemiparesis, dysarthria, and altered mental status were observed. Neurological consultation and computed tomography (CT) angiography of the head revealed an AIS with proximal occlusion of the M2 segment of the right middle cerebral artery (Fig. 1). Thrombolysis with tenecteplase 0.25 mg/kg (3 ml) was performed. Within 1 h, significant clinical improvement was observed with almost complete recovery of motor function on the paralyzed side.

**Figure 2:**
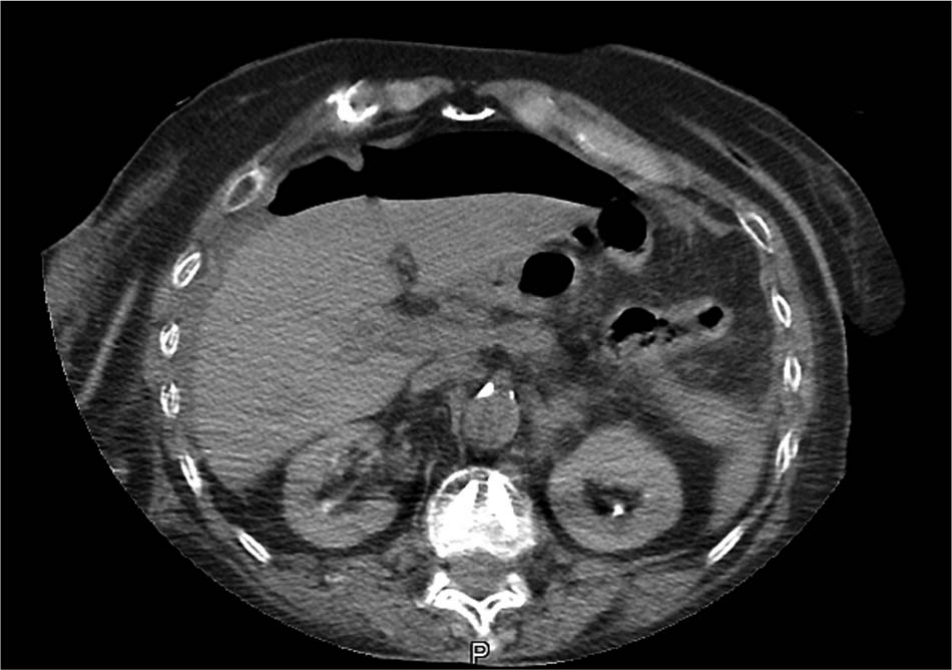
CT image of abdominal free gas.

Due to persistent abdominal pain, a computed tomography scan of the abdomen was performed ~12 h after thrombolysis. The results showed a perforated gastric ulcer with free gas in the abdomen (Fig. 2). Emergency laparotomy revealed a perforated duodenal ulcer (PDU), just posterior to the pylorus, with total purulent peritonitis. Surgical treatment included excision of the ulcer and Mikulitz pyloroplasty with a Cellan-Jones patch. The procedure was completed with peritoneal lavage and drain placement.

In ICU on postoperative day 2, atrial fibrillation was noted, which was treated with amiodarone infusions, restoring sinus rhythm. On postoperative day 5, the patient experienced an epileptic seizure, initially treated with valproate 1000 mg intravenously, then 750 mg orally daily. On postoperative day 7, a minor surgical site infection was found, but it was managed conservatively with removal of several skin staples. On postoperative day 12, the patient was discharged and referred to a rehabilitation center.

On follow-up, she demonstrated full range of motion in all limbs only during motor tests with mild paresis of the left hand, mainly at the wrist, and no gastrointestinal complaints.

## Discussion

Patients with PDU are typically a cohort with a high mortality rate, reaching 30% [1]. AIS in PDU is a rare complication and the available data are limited. Thrombolysis for the treatment of AIS has emerged as an alternative to invasive procedures, with no long-term increase in deficits observed [2].

Tenecteplaze is a genetically modified variant of tissue plasminogen activator (tPA) with biphasic clearance and a terminal half-life of up to 130 min [3, 4], emerging as an alternative to Alteplase [5]. Tenecteplaze exhibits 15-fold higher fibrin specificity and 80-fold reduced binding to plasminogen activator inhibitor [6, 7], thus increasing its efficacy and showing more favorable drug characteristics [8]. Some studies have shown that Tenecteplaze exhibits equivalent or even better results than Alteplase in the treatment of IS with large vessel occlusion [2, 9].

In our case, the initial evaluation did not reveal any absolute contraindications for systemic thrombolysis, but only an increased risk of cerebral hemorrhage due to an untreated aneurysm. After the bolus, the stroke symptoms resolved within 1 h with a residual mild paresis of the left arm—NIH Stroke Scale 2, modified Rankin Scale score 3. A follow-up CT scan 24 h after thrombolysis showed no acute ischemic areas or bleeding in the brain.

An abdominal CT scan performed for persistent abdominal pain revealed free abdominal gas, and laparotomy revealed a PDU. Various techniques, both open and minimally invasive, can be used to repair a PDU [10, 11]. The proximity of the ulcer to the duodenum was the factor that led us to choose Mikulitz pyloroplasty for the patient. It is well established that systemic thrombolysis may increase the risk of postoperative bleeding at the surgical site and may be used outside the approved indications, but only after a risk–benefit assessment [3]. In this case, we chose to perform open reconstruction because of uncertainty regarding the timing of perforation and prior administration of thrombolysis. There were also concerns about bleeding control. The operation was uneventful and hemostasis was easily achieved. The estimated blood loss was <50 cc.

No change in neurological status was observed postoperatively. Although there were postoperative complications (atrial fibrillation, seizure, and minor surgical intervention at the surgical site), the patient recovered well and was able to overcome the condition with minimal sequelae.

The main limitation of this case is the fact that there are no relevant articles and our findings cannot be compared with results from similar studies.

## Conclusion

This case represents an unconventional, simultaneous approach to the treatment of both initial conditions—the combination of AIS, PDU, and postoperative complications. Both methods of treatment—venous thrombolysis and surgical procedure—can be successfully applied in well-selected cases.
